# Determining the optimal strategies to achieve elimination of transmission for *Schistosoma mansoni*

**DOI:** 10.1186/s13071-022-05178-x

**Published:** 2022-02-14

**Authors:** Klodeta Kura, Diepreye Ayabina, T. Deirdre Hollingsworth, Roy M. Anderson

**Affiliations:** 1 London Centre for Neglected Tropical Disease Research, London, UK; 2 Department of Infectious Disease Epidemiology, School of Public Health, Faculty of Medicine, St Mary’s Campus, Imperial College London, London, UK; 3 MRC Centre for Global Infectious Disease Analysis, London, UK; 4 Big Data Institute, Li Ka Shing Centre for Health Information and Discovery, University of Oxford, Oxford, OX3 7LF UK; 5 The DeWorm3 Project, The Natural History Museum of London, London, UK

**Keywords:** Schistosomiasis, Mathematical models, Individual-based stochastic models, Mass drug administration, School-based treatment, Community-wide treatment, Elimination of transmission, Elimination as a public health problem

## Abstract

**Background:**

In January 2021, the World Health Organization published the 2021–2030 roadmap for the control of neglected tropical diseases (NTDs). The goal for schistosomiasis is to achieve elimination as a public health problem (EPHP) and elimination of transmission (EOT) in 78 and 25 countries (by 2030), respectively. Mass drug administration (MDA) of praziquantel continues to be the main strategy for control and elimination. However, as there is limited availability of praziquantel, it is important to determine what volume of treatments are required, who should be targeted and how frequently treatment must be administered to eliminate either transmission or morbidity caused by infection in different endemic settings with varied transmission intensities.

**Methods and Results:**

In this paper, we employ two individual-based stochastic models of schistosomiasis transmission developed independently by the Imperial College London (ICL) and University of Oxford (SCHISTOX) to determine the optimal treatment strategies to achieve EOT. We find that treating school-age children (SAC) only is not sufficient to achieve EOT within a feasible time frame, regardless of the transmission setting and observed age–intensity of infection profile. Both models show that community-wide treatment is necessary to interrupt transmission in all endemic settings with low, medium and high pristine transmission intensities.

**Conclusions:**

The required MDA coverage level to achieve either transmission or morbidity elimination depends on the prevalence prior to the start of treatment and the burden of infection in adults. The higher the worm burden in adults, the higher the coverage levels required for this age category through community-wide treatment programmes. Therefore, it is important that intensity and prevalence data are collected in each age category, particularly from SAC and adults, so that the correct coverage level can be calculated and administered.

**Graphical Abstract:**

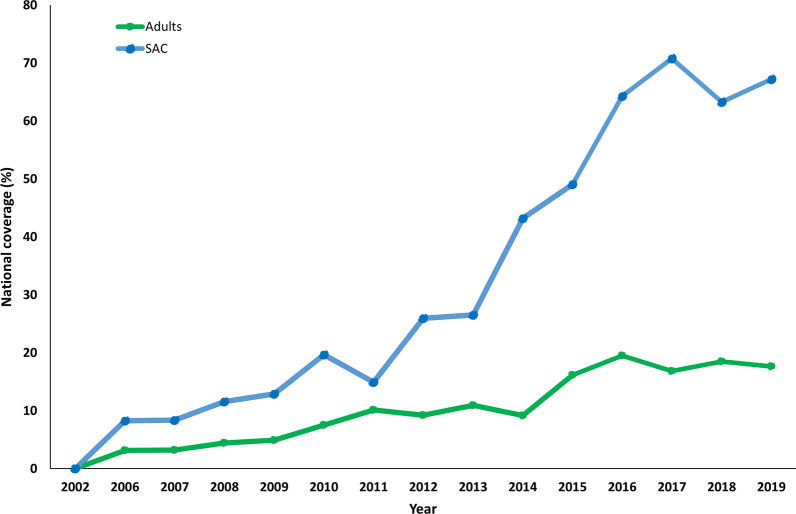

## Background

Schistosomiasis is an intestinal or urogenital disease caused by infection with *Schistosoma* trematode species, predominantly *Schistosoma mansoni*, *S. japonicum* and *S. haematobium* [1]. An individual becomes infected when the cercarial larval forms of the parasite (released by the intermediate host, which are various species of freshwater snails) penetrate the skin during contact with contaminated water. Symptoms of intestinal schistosomiasis can vary from abdominal pain, blood in stool, diarrhoea and Katayama fever, to enlargement of the liver and spleen, fibrosis, portal hypertension and accumulation of fluid in the peritoneal cavity in most advanced stages. Urogenital schistosomiasis can cause bloody urine, kidney damage and bladder cancer in the most severe cases [2, 3].

Mass drug administration (MDA), using the drug praziquantel (PZQ), is the main form of control at present, alongside behaviour modification and improvement in sanitation. Current work on potential vaccine candidates is promising, but at an early stage at present [4–7].

According to the World Health Organization (WHO), an estimated 236 million people require MDA worldwide, of which 90% live in Africa [8]. Schistosomiasis is endemic in 78 countries, of which 51 countries have moderate to high prevalence. In 2016 nearly 24,000 people died from schistosomiasis, although in reality this number is believed to be higher, as very rarely is schistosomiasis recorded in death certificates.

Morbidity control, increasing treatment coverage to at least 75% in all school-age children (SAC; 5–14 years of age), and elimination of transmission (EOT) in the Americas and Western Pacific regions and some parts of the African Region were the targets set in the 2020 World Health Organization (WHO) roadmap [9]. To achieve these targets, WHO recommended treatment guidelines based on the baseline prevalence of infection in SAC [10]. These guidelines focused on treating SAC, as they are more likely to be infected, but inclusion of adults (≥ 15 years old) at risk of infection through occupation and/or living in high-transmission areas has also been recommended.

The fifth progress report assessing progress on the goals set in the London Declaration has scored schistosomiasis red in 2016 as there was no significant measurable progress towards the elimination target [11].

**Fig. 1:**
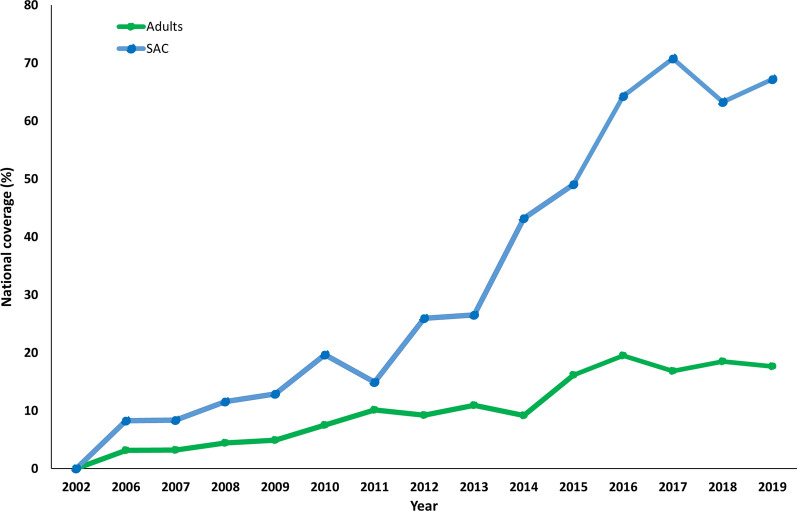
National coverage (for SAC and adults) in endemic regions as reported by the World Health Organization (WHO) preventive chemotherapy and transmission control (PCT) databank [35]

However, there has been good progress since 2016 in SAC MDA coverage (Fig. 1), partly due to the donation of 250 million PZQ tablets annually by Merck & Co. through WHO, primarily for SAC [8]. There has been low coverage in adults over the years, with figures not exceeding 20% coverage for any given country (Fig. 1). As previous research has shown, high coverage of adults is needed in many moderate- to high-transmission settings to achieve elimination as a public health problem (EPHP) [6, 12–14].

In January 2021, WHO issued the 2021–2030 roadmap for neglected tropical diseases (NTDs) [8]. The goal for schistosomiasis is EPHP, to be reached by 2023 in 49 countries, by 2025 in 69 countries and by 2030 in 78 countries (Table 1). This is to be achieved by reducing the heavy-intensity (eggs per gram ≥ 400) prevalence in SAC to less than 1%.

**Table 1:** WHO 2030 targets, sub-targets and milestones for EPHP, achieved when the heavy-intensity prevalence in SAC reduces to less than 1% [8]

Indicator	2023	2025	2030
Number of countries validated for EPHP	49/78 (63)	69/78 (88%)	78/78 (100%)
Number of countries where absence of infection in humans has been achieved	10/78 (13%)	19/78 (24%)	25/78 (32%)

The end goal for schistosomiasis is interruption of transmission, achieved when the incidence of new infections reduces to zero. The WHO roadmap suggests this should be reached by 2030 in 25 countries.

In previous work [13], we have explored the strategies in the design of MDA programmes needed to be adopted to achieve and sustain the elimination of schistosomiasis as a public health problem. This past work has focused on the MDA coverage required across all child and adult age groups to achieve specified targets by a defined year.

In this present paper, we analyse whether it is possible to achieve the interruption of transmission by exploring various treatment strategies.

## Methods

In this paper, we use two individual-based stochastic models developed independently by Imperial College London (ICL) and the University of Oxford (SCHISTOX). Details of the two transmission dynamics models have been published previously [15–19]. Both models have similar processes, except for one important difference. The ICL model assumes that the number of eggs produced is a non-linear function (density-dependent egg production) of the female worm burden assuming monogamous sexual reproduction. In contrast, SCHISTOX assumes that the number of eggs produced is proportional to the number of worm pairs (male and female worms). Both models consider a single shared environmental reservoir of infection and collapse the details of the dynamics of the snail intermediate host and the two larval stages into the expression for the basic reproduction number, R_0_, due to the huge differences between the lifespan of the different life-cycle stages (hours for miracidia and cercaria, days to weeks for infected snails, and 3.5–10.5 years for the adult parasite in humans). The two models have been calibrated to produce the same baseline settings by varying the basic reproduction number, R_0_, in the ICL model, and the overall contact rate in the SCHISTOX model. Existing estimates for the basic reproduction number for schistosomiasis are typically in the range of 1–4 [15–19]. In our simulations, to analyse the impact of MDA in reducing the prevalence of disease, we assume a population of 500 individuals without migration and random coverage at each round of treatment, e.g., at each MDA round a new random defined fraction of the target group is treated. Persistent non-compliance is not addressed hence the predictions may be on the optimistic side. We also consider two age–intensity profiles of infection, corresponding to low and high adult burden of infection, achieved by varying the age-specific contact rates (Table 2). A description of all parameters used, and their values are given in Table 2.

**Table 2:** Parameter values used for *S. mansoni* (for both ICL and SCHISTOX model)

Parameter	Value	References
Fecundity	0.34 eggs/female/sample	[1, 36, 37]
Aggregation parameter	0.04 for low-prevalence settings; 0.24 for high-prevalence settings	[1, 38]
Density-dependent fecundity	0.0007/female worm	[1, 39]
Worm lifespan	5.7 years	[1, 16, 40]
Drug efficacy	86.3%	[41]
Low adult burden setting: age-specific contact rates for 0–4, 5–9, 10–15, 16 + years old	0.01, 1.2, 1, 0.02	[39]
High adult burden setting: age-specific contact rates for 0–4, 5–11, 12–19, 20 + years old	0.01, 0.61, 1, 0.12	[39]
Prevalence of infection	Percentage of population having > 0 eggs per gram [epg]	-
Heavy-intensity infection prevalence	Percentage of population having ≥ 400 epg	[42]

### Scenarios simulated

In our simulations, we consider low (SAC prevalence < 10%), moderate (SAC prevalence 10–50%) and high (SAC prevalence ≥ 50%) baseline prevalence settings, with a low or high adult burden of infection (Table 1). These reflect different pristine transmission settings as defined by the magnitude of the basic reproductive number, R_0_.

Previous mathematical work [13] has shown that it is possible to achieve and sustain the EPHP goal by adopting various MDA treatment strategies. The probability of achieving and maintaining this goal depends on the baseline prevalence prior to treatment, the precise form of the age–intensity profile of infection and the strategy adopted in terms of MDA coverage and treatment frequency.

Building on this past published research, we explore whether we can achieve EOT by considering the following scenarios:

1. Can we move from EPHP to EOT? In this scenario, we explore whether we can move towards EOT after achieving EPHP. We continue the MDA programme (once the EPHP is reached) by giving PZQ annually to 75% of SAC (the WHO-recommended treatment coverage) only for up to a further 10 years. If the EOT goal has not been met, we increase the SAC coverage or/and include adults in the treatment programme so that the goal can be met within 10 years.
2. Can we achieve EOT within 15 years of the start of the programme (from baseline) by intensifying the treatment strategies with more frequent treatment and higher MDA coverage levels? In this scenario, we do not have the EPHP goal as a sub-target. We consider various MDA coverage combinations in SAC and adults and find whether the EOT target can be achieved.

At the end of the programme, we evaluate the population prevalence (i.e., across all age groups) to determine whether the EOT has been met. We consider the EOT to be achieved when the overall prevalence is reduced to less than 1% by single Kato–Katz on two samples per individual using a sample size of 200 individuals from all age categories, regardless of the burden of infection in adults [20].

Each scenario is run 500 times and the EOT goal is considered achieved when at least 90% of simulations are below 1%.

## Results

### Can we move from EPHP to EOT?

Here we present results and analyse what is required to achieve EOT after achieving EPHP. Table 3 presents the results for low adult burden of infection and Table 4 presents results for the example of a high adult burden of infection.

**Table 3:** Model-recommended treatment strategies for achieving EPHP and EOT (after the achievement of EPHP) for low adult burden of infection. Results for low-transmission settings are generated using the ICL model

Prevalence in SAC prior to treatment	Model	Model-recommended treatment strategy for achieving EPHP [13]	Model-recommended treatment strategy for achieving EOT after the achievement of EPHP
Low (< 10%)	ICL	75% SAC for 2–3 years	75% SAC and 30% adults for 5–6 years
Moderate (10–50%)	ICL	75% SAC for 4 years	75% SAC and 40% adults for 8–9 years
	SCHISTOX	75% SAC for 5 years	75% SAC and 40% adults for 6–7 years
High (≥ 50%)	ICL	75% SAC for 4–10 years (baseline prevalence below 67% SAC + 56% adults) 90% SAC and 45% adults for 10 years (baseline prevalence: 71% SAC + 60% adults)	75% SAC and 50% adults for 8–10 years (baseline prevalence below 67% SAC + 56% adults) 75% SAC and 50% adults for 5 years (baseline prevalence: 71% SAC + 60% adults)
	SCHISTOX	75% SAC for 5–10 years (baseline prevalence below 76% SAC + 42% adults) 90% SAC and 45% adults for 8 years (baseline prevalence: 79% SAC + 49% adults)	75% SAC and 50% adults for 7–8 years (baseline prevalence below 76% SAC + 42% adults) 75% SAC and 50% adults for 5 years (baseline prevalence: 79% SAC + 49% adults)

**Table 4:** Model-recommended treatment strategies for achieving EPHP and EOT (after the achievement of EPHP) for high adult burden of infection. Results for low-transmission settings are generated using the ICL model

Prevalence in SAC prior to treatment	Model	Model-recommended treatment strategy for achieving EPHP	Model-recommended treatment strategy for achieving EOT after the achievement of EPHP
Low (< 10%)	ICL	75% SAC for 2–4 years	75% SAC and 30% adults for 8–9 years
Moderate (10–50%)	ICL	75% SAC for 5–7 years	75% SAC and 55% adults for 9–10 years
	SCHISTOX	75% SAC for 6–8 years	75% SAC and 55% adults for 7 years
High (≥ 50%)	ICL	90% SAC and 45% adults for 5–6 years (baseline prevalence below 60% SAC + 63% adults) 95% SAC and 85% adults for 10 years (baseline prevalence: 71% SAC + 72% adults)	75% SAC and 55% adults for 7–8 years (baseline prevalence below 60% SAC + 63% adults) 85% SAC and 75% adults for 9 years (baseline prevalence: 71% SAC + 72% adults)
	SCHISTOX	90% SAC and 45% adults for 4–8 years (baseline prevalence below 76% SAC + 64% adults) 95% SAC and 85% adults for 8 years (baseline prevalence: 79% SAC + 72% adults)	75% SAC and 55% adults for 6–7 years (baseline prevalence below 76% SAC + 64% adults) 85% SAC and 75% adults for 8 years (baseline prevalence: 79% SAC + 72% adults)

### Low-transmission settings

In low-transmission settings with a low adult burden of infection (Table 3), we find that annual treatment of 75% of SAC alone can achieve the EPHP goal within 3 years. Continuing MDA with the same coverage for another 5–6 years (depending on the baseline prevalence) can achieve the EOT with high probability. Therefore, up to 9 years is required to achieve the EOT goal from the start of the programme (at baseline).

For high adult burden of infection (Table 4), as expected, it takes longer for these goals to be achieved. The EPHP can be met within 4 years of treatment, while the EOT can be achieved within 9 years after the EPHP has been met. For both these goals, coverage of 75% of SAC has been used. Thus, in total it takes up to 13 years for the EOT goal to be met from the start of the MDA programme with high annual coverage of SAC.

### Moderate-transmission settings

For low adult burden of infection (Table 3), our models predict that treating 75% of SAC can achieve the EPHP goal within 5 years of treatment. After the EPHP goal has been achieved, we can restart the programme. We find that treating 75% of SAC only cannot achieve the EOT goal. However, including adults in the treatment programme can increase the probability of achieving this goal. We find that using coverage of 75% among SAC and 40% among adults can achieve the EOT goal within 9 years.

For high adult burden of infection (Table 4), the EPHP is achieved within 8 years by following the WHO-recommended treatment strategy of 75% SAC. We need to continue the programme (after the achievement of EPHP) for up to 10 more years and use a coverage of at least 75% SAC and 55% adults to achieve the EOT goal.

### High-transmission settings

For low adult burden of infection (Table 3) and SAC baseline prevalence less than 67%, the ICL model predicts that administering MDA to 75% of SAC can achieve the EPHP goal within 10 years. An extra 10 years and a coverage of 75% of SAC and 50% adults might be needed to achieve EOT. For baseline prevalence above this threshold (67%), we need to increase the SAC coverage and include adults in the treatment programme to achieve EPHP within 10 years. Administering MDA to 90% of SAC and 45% of adults can achieve this goal within 10 years. After this point, another 5 years and a coverage of 75% among SAC and 50% among adults are required to achieve the EOT goal.

In contrast, the SCHISTOX model predicts that treating 75% of SAC can achieve the EPHP goal within 10 years for baseline prevalence less than 76%. An extra 5 years are required to achieve the EOT. For baseline prevalence above this threshold, 8 years are required to achieve EPHP and an extra 5 years to achieve EOT.

For high adult burden of infection (Table 4), the ICL model predicts that EPHP can be achieved by treating 90% of SAC and 45% of adults annually for up to 6 years if the baseline SAC prevalence is below 60%. After achieving EPHP, the EOT can be achieved by treating 75% of SAC and 55% of adults annually for up to 8 years. Thus, it takes up to 14 years to achieve the EOT since the start of the MDA programme (at baseline).

The SCHISTOX model, predicts that EPHP can be achieved by treating 90% of SAC and 45% of adults annually for up to 8 years if the baseline SAC prevalence is below 76%. After achieving EPHP, the EOT can be achieved by treating 75% of SAC and 55% of adults annually for up to 7 years. Therefore, using the SCHISTOX model, it takes up to 15 years to achieve the EOT target from the start of the MDA programme.

For baseline SAC prevalence above these thresholds, intensified treatment is needed, such as higher coverage of SAC and adults.

### Intensive treatment strategies

Here we do not have the elimination of a public health problem as a sub-target. This strategy can be considered in low-transmission settings where the only goal from the start of the treatment programme is EOT. For example, the ongoing Geshiyaro Project in southern Ethiopia is assessing various treatment strategies to achieve EOT for schistosomiasis and soil-transmitted helminths (STH) within 6 years of treatment [21].

**Fig. 2:**
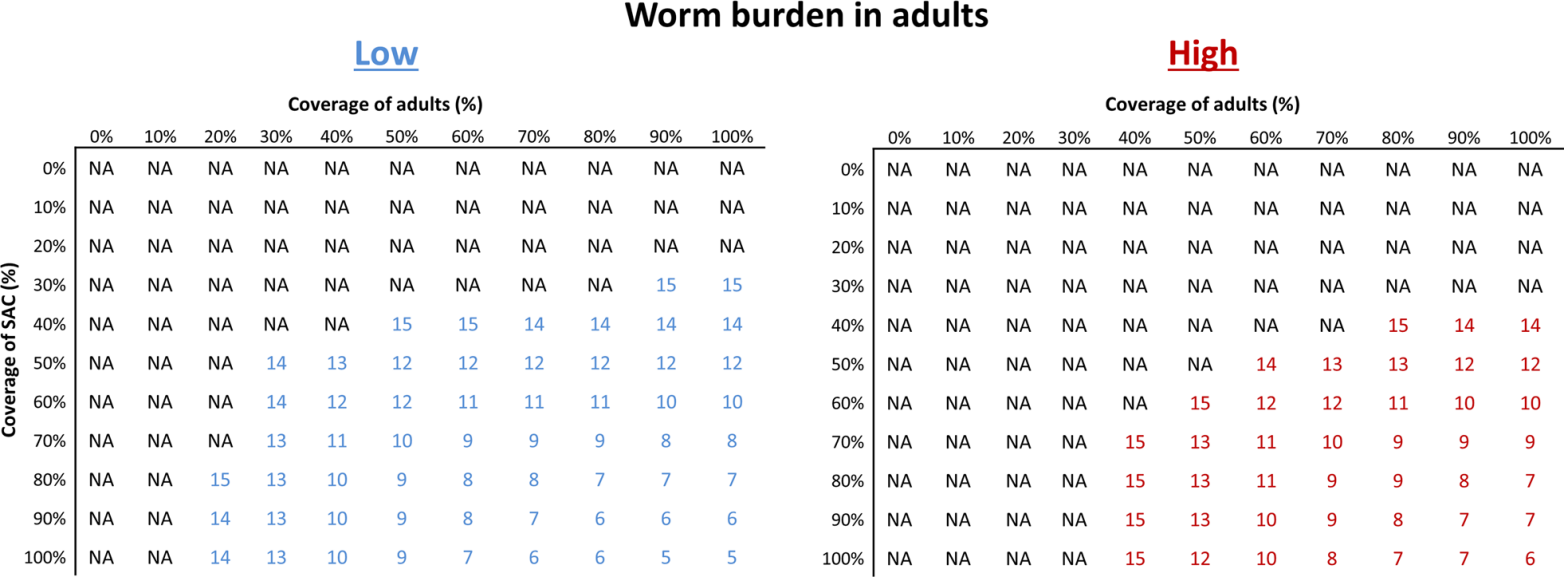
Number of years of annual treatment to achieve elimination of transmission of *S. mansoni* as a function of coverage of SAC versus adults for low-transmission settings and low to high worm burden of infection in adults in low-transmission settings (baseline = 9%). NA: not achievable within 15 years of annual MDA. Results shown are generated using the ICL model

In Fig. 2, we have presented results on the number of years of annual treatment to achieve EOT of *S. mansoni* as a function of coverage of SAC versus adults for low-transmission settings and low/high worm burden of infection in adults.

These results show that treating SAC only cannot achieve EOT within 15 years of treatment, regardless of the burden of infection in adults. This is due to the fact that the untreated adults continue to contribute to the reservoir of infection, making it impossible to break transmission. Community-wide treatment (SAC and adults) can achieve EOT for low and high adult burden of infection. The number of years of annual treatment depends on the coverage level for both SAC and adults and the worm burden in adults. Using intensive treatment strategies (100% of SAC and 100% of adults) can achieve EOT within 5 and 6 years for low and high adult burden of infection, respectively. Less intensive strategies, such as treating at least 70% of SAC and 50% of adults can achieve the EOT within 10 years of annual treatment if the burden of infection in adults is low. For a high burden of infection in adults, this target can be met within the same time, by treating 70% of SAC and 70% of adults.

## Discussion

The 2030 goal for schistosomiasis is EPHP, to be achieved in 78 countries (Table 1), and the end goal is interruption of transmission, achieved when the incidence of infection reduces to zero, to be achieved by 2030 in 25 countries. In this study, we explore various treatment strategies and assess whether it is possible to eliminate schistosomiasis by using MDA alone, and if so, what treatment coverage is required for SAC and adults.

We find that the outcome depends on the transmission setting (low and high R_0_ values), worm burden in adults, MDA coverage level and the length of the annual treatment MDA programme. For low-transmission settings with a low adult burden of infection, treating SAC only cannot achieve the EOT goal (Table 3). However, we can achieve this goal within 9 years if we initially treat 75% of SAC annually for up to 3 years (time to EPHP) and then treat 75% of SAC plus 30% of adults for the next 6 years. Using more intensive strategies such as treating 90% of SAC and 80% of adults annually can achieve EOT within 6 years of the MDA programme (Fig. 2).

For low-transmission settings with a high adult burden of infection (Table 4), like results for low adult burden of infection, we cannot achieve EOT by treating SAC only. However, we can achieve this goal within 13 years if we initially treat 75% of SAC annually for up to 4 years (time to EPHP) and then treat 75% of SAC plus 30% of adults for the next 9 years. Again, more intensive strategies can achieve this goal within a shorter time frame (Fig. 2). It should be noted here that results for low-transmission settings are generated using the ICL model only, as the SCHISTOX model typically cannot reproduce endemic prevalence less than about 12%.

In moderate-transmission settings with a low adult burden of infection, treating 75% of SAC and 40% of adults annually, after the achievement of EPHP, can eliminate transmission within 9 years. For high adult burden of infection, analysis suggests that treating 75% of SAC and 55% of adults annually after the EPHP has been met can achieve the EOT within 10 years.

In high-transmission settings, regardless of the burden of infection in adults, there is a cut-off prevalence where more intensive strategies, such as higher coverage of SAC and adults, are required to eliminate transmission. This threshold varies with the age–profile of infection and across the two models used in this study.

It should be noted that, even when the EPHP goal has been achieved, higher coverage levels amongst SAC and adults are required to move towards EOT. Specifically, the coverage amongst SAC varies between 75 and 95% and the coverage amongst adults varies between 30 and 75%, depending on the baseline prevalence and the worm burden in adults. This is because, although the heavy-intensity prevalence in SAC is reduced to less than 1% (for the EPHP), the prevalence in SAC and adults remains high. A large reduction in the prevalence of heavy-intensity infection does not necessarily correspond to a large reduction in the prevalence of infection. After the EPHP has been achieved, there might be light- to moderate-intensity infections in SAC and light- to heavy-intensity infections in the non-SAC age categories. Therefore, stopping MDA after this goal has been achieved will lead to a resurgence in infection across all age classes [20].

Even though the EOT goal is achievable for all transmission settings and levels of infection in adults (with prolonged programmes), in some settings it might not be feasible to achieve high coverage levels, particularly amongst adults due to limited PZQ availability and hard-to-reach adult individuals.

An important consideration is that preschool-age children (pre-SAC) have not been included in the treatment programme, due to the absence of clinical data on the drug safety and efficacy for young children [22]. A phase III study is underway to evaluate the efficacy and safety of a new PZQ formulation, developed by the Paediatric Praziquantel Consortium [23]. This new formulation is a small tablet with an acceptable taste for this age category. However, our results show that even when excluding pre-SAC from the treatment programme, transmission elimination can be achieved. Inclusion of this age group in MDA programmes can shorten the time to EOT as well as reduce the coverage level required in SAC and adults.

Consideration of the impact of animal reservoirs and understanding the importance of potential zoonotic species in transmission within human communities will become increasingly important as infection in human populations is reduced to low levels and nears potential elimination. The schistosomiasis transmission models will require extension to multi-host frameworks to quantify the risk of animal reservoirs sustaining transmission in human communities and to help inform what and where complementary ‘One Health’ interventions may be required [24, 25].

The prevalence of infection in our study is measured using a single Kato-Katz on two samples per individual [26]. However, for programmes aiming at EPHP or EOT, the Kato-Katz diagnostic technique suffers from low sensitivity to detect infection at very low prevalence [27]. Identifying low-prevalence endemic settings and tackling low-prevalence resurgent infection in post-treatment surveillance may require different diagnostic techniques. The point-of-care circulating cathodic antigen (POC-CCA) is an alternative diagnostic technique which has greater sensitivity than Kato-Katz and performs better at detecting infection in low-prevalence endemic settings [28–30].

In this work, we have assumed random adherence to treatment with a fixed MDA coverage level. However, this may not always be the case, and as a result, our predictions may be on the optimistic side. Persistent non-compliers with MDA treatment can serve as reservoirs of infection. There is a clear need for more studies of compliance patterns in PZQ MDA-treated communities. We can address this issue by collecting and recording data on individual compliance, but to date very little attention has been paid to this during monitoring and evaluation of schistosomiasis control programmes.

Schistosomiasis can be highly heterogeneous on small scales, which in combination with human movement can impact the success of the MDA by reducing the probability of elimination [31]. As programmes transition towards EOT, understanding how observed heterogeneity in endemicity patterns influences and is influenced by transmission and control measures becomes crucial. Future analysis will be extended to integrate the current transmission model with models of human movement and to determine the optimal strategy for each spatial scale (district, subdistrict and community/village).

In this paper, we have not included the impact of acquired immunity, as it is not possible to distinguish the effects of immunity from the age-dependent exposure to infection. Both are probably important determinants of the observed patterns of infection across age classes. Some level of immunity is believed to slowly build up over long periods of exposure, which can lessen the impact of MDA in achieving elimination [32–34].

As MDA does not prevent reinfection, snail control can be an important component of the current schistosomiasis elimination strategy, especially in high-transmission areas. Previous modelling by Li and colleagues [43] showed that the addition of snail control can reduce the duration of treatment required to achieve elimination. While this treatment strategy can increase the impact, the lack of efficacy data limits model predictions of its potential benefits.

Much more attention in future modelling studies should be given to the impact of MDA plus snail control in reducing the prevalence of infection.

Additional treatment interventions include improving water, sanitation and hygiene (WASH), or vaccination if one becomes available in the coming years.

## Conclusions

In conclusion, we find that treating SAC only cannot achieve EOT within a feasible time frame. However, it is possible to achieve this target if high and moderate-to-high annual treatment coverage is administered in SAC and adults.

## Data Availability

All data generated or analysed during this study are included in this published article.
